# Massive Depletion of Bovine Leukemia Virus Proviral Clones Located in Genomic Transcriptionally Active Sites during Primary Infection

**DOI:** 10.1371/journal.ppat.1003687

**Published:** 2013-10-03

**Authors:** Nicolas A. Gillet, Gerónimo Gutiérrez, Sabrina M. Rodriguez, Alix de Brogniez, Nathalie Renotte, Irene Alvarez, Karina Trono, Luc Willems

**Affiliations:** 1 Molecular and Cellular Epigenetics, Interdisciplinary Cluster for Applied Genoproteomics (GIGA) of University of Liège (ULg), Sart-Tilman, Liège, Belgium; 2 Molecular and Cellular Biology, Gembloux Agro-Bio Tech, University of Liège (ULg), Gembloux, Belgium; 3 Instituto de Virología, Centro de Investigaciones en Ciencias Veterinarias y Agronómicas, INTA, Castelar, Argentina; University of Pennsylvania School of Medicine, United States of America

## Abstract

Deltaretroviruses such as human T-lymphotropic virus type 1 (HTLV-1) and bovine leukemia virus (BLV) induce a persistent infection that remains generally asymptomatic but can also lead to leukemia or lymphoma. These viruses replicate by infecting new lymphocytes (i.e. the infectious cycle) or via clonal expansion of the infected cells (mitotic cycle). The relative importance of these two cycles in viral replication varies during infection. The majority of infected clones are created early before the onset of an efficient immune response. Later on, the main replication route is mitotic expansion of pre-existing infected clones. Due to the paucity of available samples and for ethical reasons, only scarce data is available on early infection by HTLV-1. Therefore, we addressed this question in a comparative BLV model. We used high-throughput sequencing to map and quantify the insertion sites of the provirus in order to monitor the clonality of the BLV-infected cells population (i.e. the number of distinct clones and abundance of each clone). We found that BLV propagation shifts from cell neoinfection to clonal proliferation in about 2 months from inoculation. Initially, BLV proviral integration significantly favors transcribed regions of the genome. Negative selection then eliminates 97% of the clones detected at seroconversion and disfavors BLV-infected cells carrying a provirus located close to a promoter or a gene. Nevertheless, among the surviving proviruses, clone abundance positively correlates with proximity of the provirus to a transcribed region. Two opposite forces thus operate during primary infection and dictate the fate of long term clonal composition: (1) initial integration inside genes or promoters and (2) host negative selection disfavoring proviruses located next to transcribed regions. The result of this initial response will contribute to the proviral load set point value as clonal abundance will benefit from carrying a provirus in transcribed regions.

## Author Summary

Human T-lymphotropic Virus 1 (HTLV-1) induces a persistent infection that remains generally asymptomatic. Nevertheless, in a small proportion of individuals and after a long latency, HTLV-1 infection leads to leukemia or lymphoma. Onset of clinical manifestations correlates with a persistently elevated number of infected cells. Because the vast majority of cells are infected at early stages, primary infection is a crucial period for HTLV-1 persistence and pathogenesis. Since HTLV-1 is transmitted through breast feeding and because systematic population screenings are rare, there is a lack of available samples at early infection. Therefore, we addressed this question in a closely related animal model by inoculating cows with Bovine Leukemia Virus (BLV). We show that the vast majority of cells becoming infected during the first weeks of infection and do not survive later on. We also demonstrate that the initial host selection occurring during primary infection will specifically target cells that carry a provirus inserted in genomic transcribed regions. This conclusion thus highlights a key role exerted by the host immune system during primary infection and indicates that antiviral treatments would be optimal when introduced straight after infection.

## Introduction

The deltaretrovirus genus includes human T-lymphotropic viruses (HTLVs), simian T-lymphotropic viruses (STLVs) and the bovine leukemia virus (BLV). These viruses induce a life-long persistent infection that remains generally asymptomatic (reviewed by [1]–[3]). Nevertheless, HTLV-1 and BLV cause leukemia or lymphoma in a minority of infected hosts after a long period of latency [3], [4]. Viral spread within the host uses two distinct processes. First, the infectious cycle results from virion attachment to target lymphocytes, entry of viral single-stranded RNA, reverse-transcription and integration as provirus into the host genome (also known as the infectious cycle) [5]–[7]. The second strategy of replication relies on driving cell proliferation using viral regulatory proteins such as Tax (i.e. the mitotic cycle) [8], [9]. These two viral replication routes thus generate a series of infected cell populations that are composed of numerous distinct clones (i.e. a population of cells carrying the provirus at a given site of the host genome). Animal models using experimental inoculation of squirrel monkey with HTLV-1 or sheep with BLV demonstrated that the infectious cycle dominates early infection and finishes 1 to 8 months later [10], [11]. Thereafter, the proviral load (PVL) is mainly maintained by mitotic replication of infected cells [12]–[15]. In HTLV-1 infected individuals, the majority of the infected clones are indeed relatively stable during many years [16]. Remarkably, using BLV-sheep experimental infection, it has been shown that the leukemic clone can be detected as early as one month after inoculation [17]. Thus, efficient virus replication via production of virions and infection of new target cells occurs mostly during a very short period following viral inoculation (so-called primary infection). As a consequence, the vast majority of the infected clones were created during this crucial period of primary infection.

Unfortunately, these important early times of HTLV-1 infection cannot be studied due to the paucity of available samples. Therefore, very little is known about the modes of viral replication during this primary phase of infection. For instance, what proportion of clones generated during primary infection will establish in the long term? Is there a negative selection against particular clones? And if so, is there a role of genomic integration sites in clonal selection? This is particularly important because clones are not equal regarding their proliferative potential. In HTLV-1 infected patients, the abundance of a given clone is enhanced by the integration of its provirus in an actively transcribed area of the genome [16], [18]. Furthermore, it is well described that increased proviral load (PVL) and clonal expansion of infected cells are key events during the process of leukemogenesis in HTLV-1 carriers. Indeed, HTLV-1 PVL correlates with the risk of developing adult T-cell leukemia or lymphoma (ATLL) [19] and HTLV-1 clonality of ATLL patients is characterized by the presence of massively expanded clones [12], [13], [16].

In this context, the aim of this study was to evaluate clonal evolution of the infected cells with longitudinal samples during primary infection with respect to clonal diversity and host-dependent negative selection. Due to the difficulty at accessing samples from newly HTLV-1 infected patients, we addressed this question in a closely related animal model by inoculating cows with BLV. We selected animals whose BLV PVL set point values encompassed the whole range of natural variability. We then used a newly developed method to map and quantify the insertion sites of the provirus in order to monitor the clonality of the BLV-infected cells population (i.e. the number of distinct clones and abundance of each clone) during primary infection. We demonstrated that (1) BLV initially targets transcribed regions of the genome for integration; (2) later, a massive clone selection occurs during primary infection disfavoring proviruses located nearby genes; (3) nevertheless, the abundance of the long term maintained clones benefits from the transcriptional activity of the genomic region surrounding the provirus.

## Results

### Antibody response against BLV and BLV proviral load evolution during primary infection

**Figure 1:**
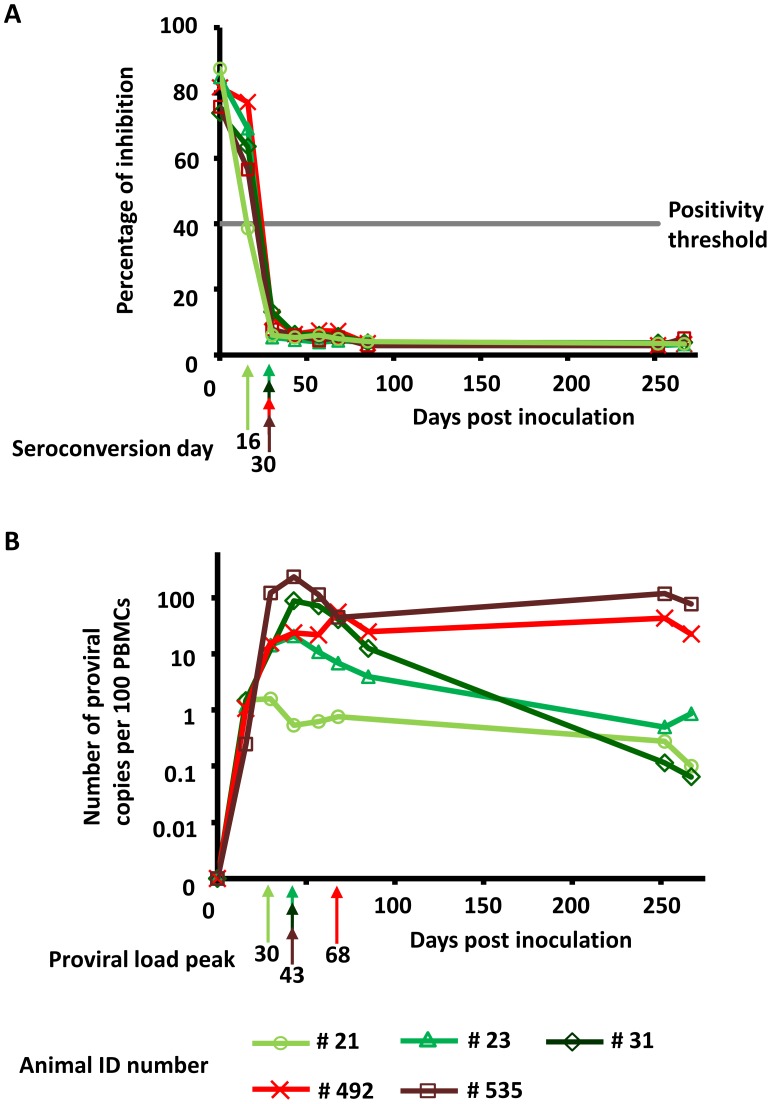
Antibody responses against BLV and BLV proviral loads. **A.** BLV was inoculated at day zero. Antibodies against BLV SU were detected with a competitive ELISA. Sero-positivity (arrows) occurred when percentage of inhibition dropped below a threshold of 40% (defined as the seroconversion day). **B.** Proviral loads (PVLs) were determined by quantitative real time PCR. Arrows indicate the date when PVL reached maximum value within each animal.

Five cows were inoculated with a cloned BLV provirus (strain 344) as described in the Materials and Methods section. Figure 1A shows that all animals developed a humoral response directed against BLV SU, the viral surface envelope protein. Anti-SU antibodies were detected at 16 and 30 days post-inoculation of animals #21 and #23/31/492/535, respectively. Since then, all animals remained sero-positive for SU antibodies.

Figure 1B shows the PVL (number of proviral copies per 100 peripheral blood mononuclear cells, PBMCs) following BLV inoculation of cows at day 0. The PVLs sharply increased with a maximal value reached between 30 to 68 days post-inoculation (dpi) (30 days for #21, 43 days for #23, #31 and #535 and 68 days for #492, arrows on figure 1B). PVL peak values differed widely among animals ranging from 1.6 proviral copies per 100 PBMCs for animal #21 up to 236 proviral copies per 100 PBMCs for animal #535. Then, the PVLs subsequently decreased and reached a level that differed widely between animals. Cows #21, #23 and #31 (green lines) presented a low PVL set point (respectively 0.2, 0.5 and 0.1 copies per 100 PBMCs at day 252) as observed in the majority of infected animals kept in herds [20]. Animals #492 and #535 (red lines) developed a very high BLV burden (respectively 43.3 and 117.6 copies per 100 PBMCs at day 252) as measured in a small proportion of infected animals ([21] and INTA experimental facilities, data not shown). None of the five animals progressed to disease within two years post inoculation.

BLV inoculation into 5 cows thus resulted in the onset of an antibody response at days 16–30 which slightly preceded the maximal proviral burden (at days 30–68). This experimental setting is thus representative of BLV infection in herd conditions.

### Polyclonal propagation during primary infection

To trace BLV clonality during primary infection, proviral insertion sites were selectively amplified, identified by high-throughput sequencing and quantified as schematized in Supporting Figure S1 in Text S1. Briefly, genomic DNA was extracted from PBMCs and sonicated. The end of the BLV 3′LTR and a fragment of bovine genomic DNA were amplified by linker-mediated PCR and the products subjected to high-throughput sequencing. Proviral insertion sites were determined by alignments of sequences downstream of the 3′ LTR (read 1; Figure S1 in Text S1). A clone was defined as a population of cells carrying the BLV provirus at a given insertion site in the bovine genome. The abundance of a clone was quantified by counting the number of different shear sites for that particular clone using read 2 alignments. A complete description of the procedure is given in the Material and Methods section.

**Figure 2:**
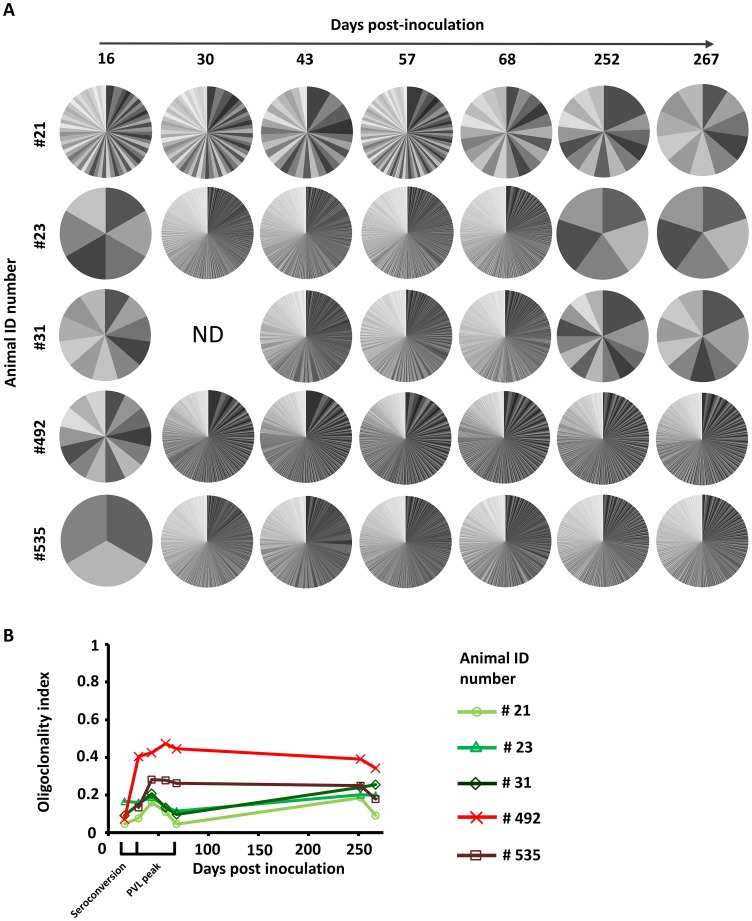
Distribution of clone abundance determined by high throughput sequencing. **A.** The pie-charts depict the distribution of clone abundance for each animal and time point. Each slice represents a particular BLV-infected clone and the size of the slice represents the relative abundance of that clone. ND: not done due to sample unavailability. Note that although the gray tones are identical at different dates, the clones are different. **B.** Oligoclonality index measures the non-uniformity of the clone abundance distribution and ranges from 0 (perfect polyclonality) to 1 (perfect monoclonality).

During primary infection, a broad range of clones were generated within each animal: from 264 in #21 to 12906 in #535 (Table S1 in Text S1). The distributions of clone abundance for each animal and time point are depicted in Figure 2A by pie charts where each slice represents the relative abundance of a given clone. Based on this data, we calculated an oligoclonality index which is a measure of the non-uniformity of the clone abundance distribution (i.e. an index close to 1 corresponds to an unevenly-distributed population composed almost of a single clone, see Supplemental Materials and Methods in Text S1). The oligoclonality index was extremely low at 16 dpi, showing that all infected clones had approximately the same abundance (Figure 2B). The oligoclonality index then rose and reached a peak value after 43 days for animals #21, #23, #31 and #535 and after 57 days for animal #492. These data thus demonstrate that early infection is characterized by a very polyclonal propagation.

### Massive depletion of BLV-infected clones after primary infection

**Figure 3:**
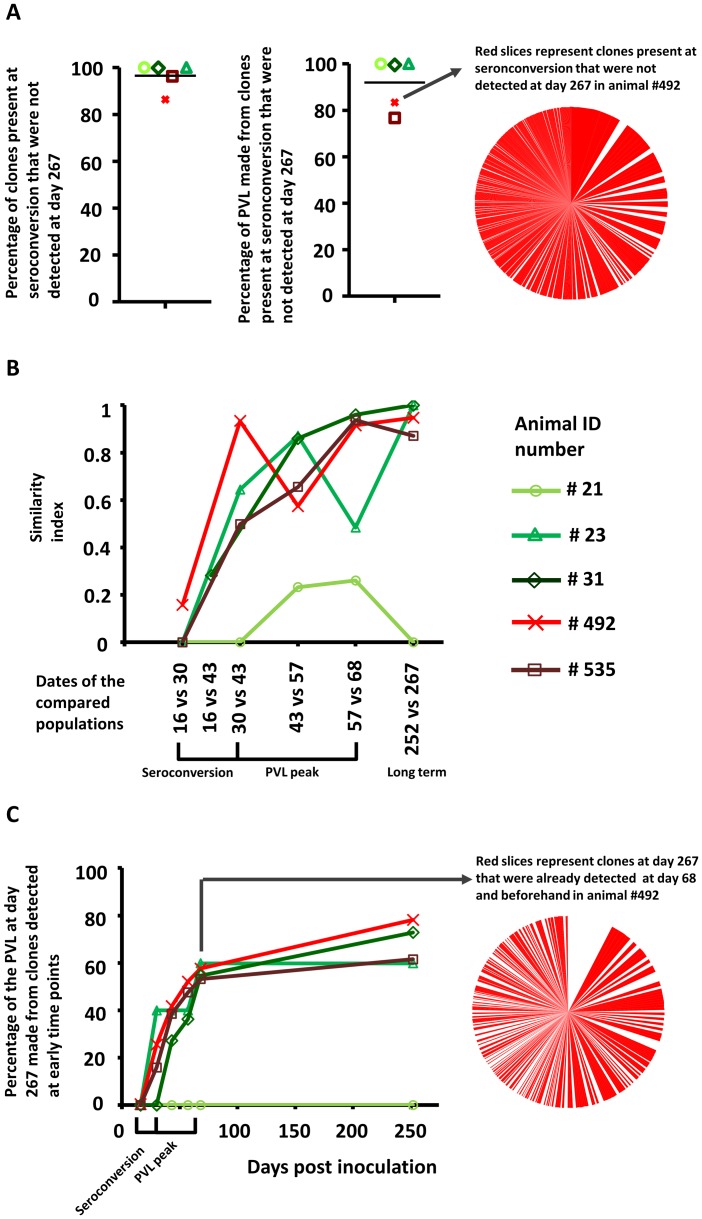
Evolution of the clonal composition. **A.** Dot-plots show the number of clones present at seroconversion that will no longer be detected at day 267. The means were indicated by the horizontal black lines. The pie-chart illustrates the massive clonal depletion observed in animal #492. Each slice represents a clone detected at seroconversion and the red ones correspond to clones that will not be detected in the long term. **B.** Similarity indexes quantify the clonal overlap between two successive populations and ranges from 0 (no clones are shared between the two populations) to 1 (the 2 populations are totally similar). The compared populations were separated by a period of about 2 weeks time. **C.** Cumulative curves showing the proportion of the PVL at day 267 that is composed from clones detected at earlier time points. The pie chart on the right illustrates the amount of clones at day 267 that were already detected at day 68 and beforehand. Each slice represents a clone detected at day 267 and the red ones correspond to clones that were detected at day 68 or before.

At later times post-infection, a massive depletion of BLV-infected clones occured (Figure 3A). The left-hand dot plot shows that, on average, 97% of the clones identified at seroconversion were no longer detected at 267 dpi. Similarly, the other dot plot shows that, on average, 92% of the proviral load at seroconversion resulted from clones that disappeared at 267 dpi. The pie chart on the right of Figure 3A illustrates this massive clonal depletion, the red slices representing the clones of #492 at dpi = 30 that were not detected in the longer term. We conclude that primary infection is thus characterized by a massive depletion of proviral clones.

### BLV replication shifts from neoinfection to clonal proliferation

To quantify the clonal overlap between two successive time points, a similarity index was calculated (see Supplemental Materials and Methods in Text S1). Figure 3B shows the evolution of the similarity index calculated between two successive populations separated by a period of two weeks. Soon after seroconversion, the similarity indexes were close to zero showing that, between 2 successive time points, the infected cell populations shared few clones. Rapidly, the similarity indexes shown on Figure 3B rose to reach values close to 1 in all animals except in #21. Infected cell populations thus stabilized in terms of clonal composition and abundance. In the long term (at dpi = 252), the similarity indexes remained close to 1 in all animals except #21. Figure 3C illustrates the kinetics of appearance of clones that were detected in the long term. It appeared that a small percentage of the long term proviral load originated from clones detected at the seroconversion period. Nevertheless, the cumulative curves rose sharply from day 30 and then reached a plateau at day 68 in all animals except #21 (Figure 3C). This shows that most clones detected in the long term originated from the PVL peak period. The pie chart on the right illustrates that a large proportion of clones established in the long term originated from the beginning of the infection. The red slices symbolize the clones present in the long term and already detected at day 68 or before. In contrast, all clones detected in the long term in animal #21 were novel, resulting in a similarity index of 0 at day 252 (Figure 3B) and a flat cumulative curve (no clone at day 267 was detected at earlier time points, Figure 3C). So, long term proviral load in animal #21 might have been maintained either by newly infected cells or by pre-existing clones not detected beforehand. It should be mentioned that the numbers of clones detected in this particular animal were low at every time point compared to the 4 other cows (see Table S1 in Text S1) due to a constantly low proviral load.

The kinetics of the appearance of long term clones and of the similarity indexes thus demonstrate that BLV propagation shifts from cell neoinfection to proliferation of preexisting clones (i.e. clonal expansion) generally soon after seroconversion.

### BLV proviral integration initially favors pol II and pol III transcribed genomic regions

**Figure 4:**
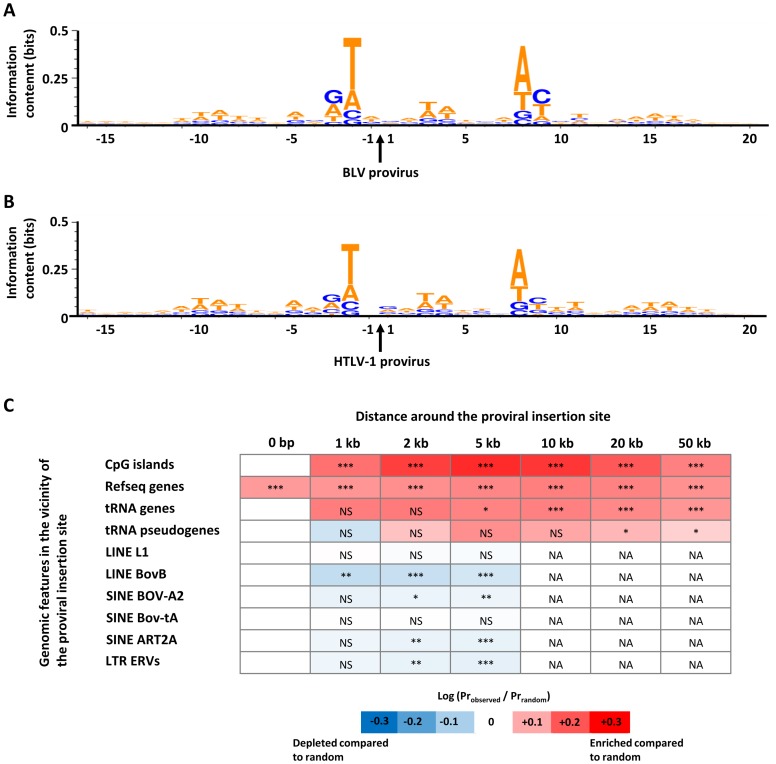
Genomic site preferences for insertion at seroconversion. **A.** Favored bovine genomic DNA sequence for BLV integration analyzed using WebLogo 3. The site of BLV integration in the target DNA sequence occurs between positions −1 and 1 with the provirus in forward orientation. Y-axis represents the information content at each target base position (perfect conservation would have a score of 2 bits). The height of the letter reflects the degree of base conservation. Data are representative for the all 5 animals. **B.** For comparison purposes, the favored human genomic DNA sequence for HTLV-1 integration is shown below. Human genomic sequences surrounding the HTLV-1 insertion sites are obtained from Gillet NA, Cook L, Laydon DJ, Hlela C, Verdonck K, et al. (2013) Strongyloidiasis and Infective Dermatitis Alter Human T Lymphotropic Virus-1 Clonality in vivo. PLoS Pathog 9(4): e1003263. doi:10.1371/journal.ppat.1003263. **C.** Genomic heat map of integration preferences at the time of seroconversion compared to the random distribution generated *in silico*. Genomic features (promoter marks, genes and interspersed repeats) analyzed are shown at the left of the heat map. For each genomic feature, the integration frequency was examined over several genomic length intervals around the proviral insertion site as shown at the top of the heat map. A colored scale is shown along the bottom of the panel with increasing shades of blue indicating depletion compared to random and increasing shades of red indicating enrichment. P-values calculated by Chi-squared test show significance of the departure from the random distribution (NS for non significant; * p<0.05; ** p<0.01; *** p<0.001; NA for non applicable). Because of the high number of interspersed repeats in the genome, all proviruses lye nearby 10 kb of such repeats and enrichment/depletion is no longer applicable (NA in the heat map). Data are representative for the all 5 animals.

The upper panel of Figure 4A shows the favored genomic consensus sequence surrounding the BLV provirus insertion site. As comparison, Figure 4B depicts the preferred insertion site of HTLV-1 in the human genome [22]. It thus appears that BLV and HTLV-1 favor highly similar genomic sequences for insertion.

BLV provirus integration did not occur randomly as illustrated on Figure 4C. Indeed, sequence analyzes demonstrate that BLV proviruses initially inserted more frequently than expected by chance inside Refseq genes (Figure 4C, second row and first column: 0 bp). Insertion was also favored nearby CpG islands, Refseq genes, tRNA genes and tRNA pseudogenes (Figure 4C, first 4 rows of the heat map). These different DNA sequences are associated with transcribed regions of the genome. Indeed, CpG islands are genomic regions with relatively high CG content generally associated with promoters [23]. Genes, whose promoters are rich in CpG sequences, tend to be expressed in most tissues (housekeeping genes). On the other hand, tRNA (transfer RNA) genes are constitutively transcribed by RNA polymerase III (pol III) unlike mRNA coding genes (which are like Refseq genes) transcribed by RNA polymerase II (pol II). Finally, tRNA pseudogenes are tRNA-derived repeats that no longer produce functional tRNA but that are still associated with polIII activity [24].

We conclude that BLV initial integration favors pol II and pol III transcribed regions of the genome.

In contrast, BLV disfavors interspersed repeats such as LINE, SINE and ERVs (Figure 4C). These elements are the largest class of sequences in mammalian genomes, making for about 50% of their total length. Most common repeats are retrotransposons that can replicate and reinsert in another site of the genome. Nevertheless, transposition does not occur in somatic cells where retrotransposons are silenced, thereby preventing insertional mutagenesis. Retrotransposons can be divided in several classes with notably SINE (Short Interspersed Nuclear Element), LINE (Long Interspersed Nuclear Element) and LTR-ERVs (Endogenous RetroVirus that are also retrotransposons with Long Terminal Repeats, LTR). Figure 4C demonstrates that insertion occurred less frequently than expected nearby retrotransposons LINE BovB, SINE BOV-A2, SINE ART2A and LTR ERVs.

We conclude that BLV initial integration disfavors transcriptionally silenced regions of the genome.

### Negative selection against proviruses located nearby transcribed regions during primary infection

**Figure 5:**
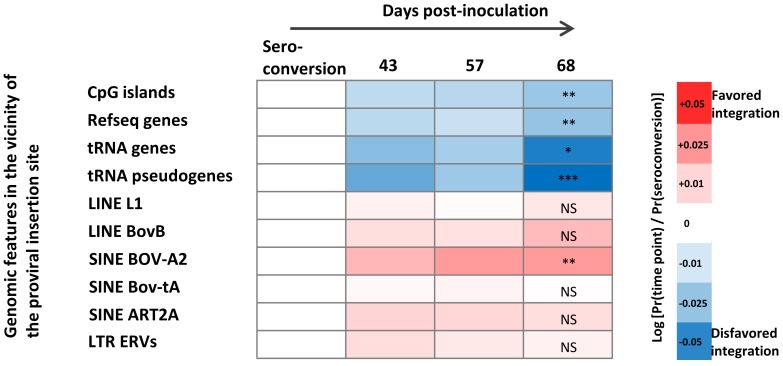
Evolution of provirus environment during primary infection. Genomic heat map of integration preferences compared to the distribution observed at the time of seronconversion. Genomic features analyzed are shown to the left of the heat map. Gene and CpG proximity was calculated with a genomic interval of 50-values are calculated by Chi-squared test for trend and show significance of the increasing or decreasing trend from the seroconversion date to day 68 post-inoculation. Data are representative for the all 5 animals.

Figure 5 illustrates the characteristics of the genomic integration site during clonal evolution from seroconversion up to day 68. The proviral environment at seroconversion serves as reference point (Figure 5, first column of the heat map). The proportion of clones carrying a provirus located next to CpG islands, Refseq genes, tRNA genes and pseudogenes significantly decreased from the seroconversion date up to 68 days after inoculation (Figure 5, first 4 lanes). In contrast, there was a trend to increase the proportion of clones containing a provirus next to genomic repeats (Figure 5, six last lanes: LINE, SINE and ERVs). The proviral environment of clones detected at days 252 or 267 was not statistically different from that observed at day 68 (data not shown).

We conclude that massive depletion of clones during primary infection is characterized by a preferential selection against proviruses inserted in transcribed regions.

### The abundance of established clones benefits from a provirus inserted in a transcribed genomic environment

**Figure 6:**
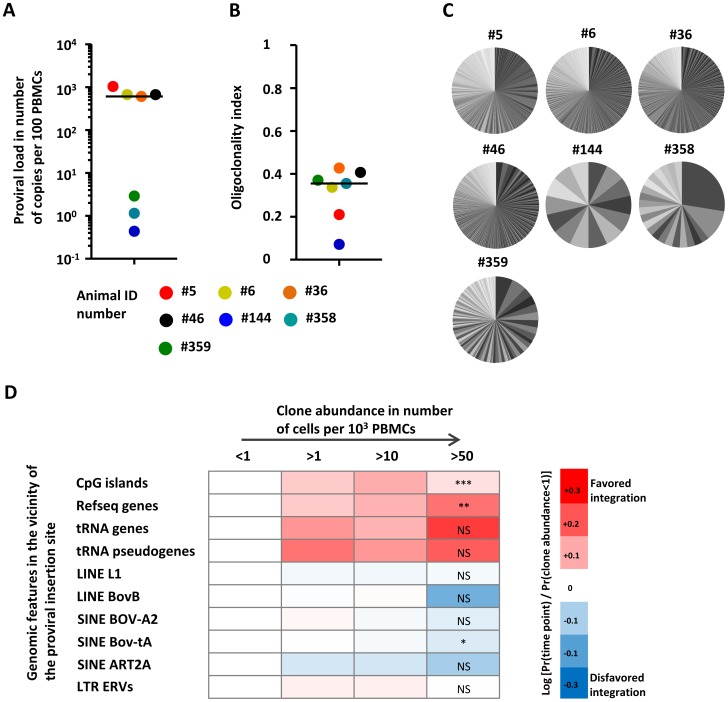
Evolution of provirus environment of clones with increasing abundance in long term infected animals. **A.** Proviral load in long-term infected animals (i.e. infected for more than 2 years). Median is indicated by the horizontal black line. **B.** Oligoclonality index in the long-term infected animals. Median is indicated by the horizontal black line. **C.** The pie-charts depict the distribution of clone abundance for each animal. The size of each slice in the pie-charts represents the relative abundance of each BLV-infected clone. Note that although the gray tones are identical between different animals, the clones are different. **D.** Genomic heat map of integration preferences compared to the distribution observed in the lowest abundance clones (clone abundance less than 1 cell per 1000 PBMCs). Genomic features analyzed are shown to the left of the heat map. Gene and CpG proximity was calculated with a genomic interval of 50 kb. An interval of 5 kb was used for interspersed repeats. For each genomic feature, the integration frequency was examined for clones having a given abundance as shown at the top of the heat map. A colored scale is shown along the right hand side of the panel with increasing shades of blue indicating depletion compared to the distribution of the lowest abundance clones and increasing shades of red indicating enrichment. In the column at the highest clone abundance (clone abundance more than 50 cells per 1000 PBMCs), p-values are calculated by Chi-squared test for trend and show significance of the increasing or decreasing trend from the smallest clones. Data are representative for all 7 animals.

Finally, we characterized the genomic environment of the proviruses that thrived in the long term. Therefore, we analyzed clonality in a series of cows infected for more than 2 years and harboring a wide range of PVL (Figure 6A). These animals presented a polyclonal distribution of BLV-infected cells (Figure 6B). The numbers of detected clones per animal are shown in Table S2 in Text S1. The distributions of clone abundance for each animal are depicted in Figure 6C. Figure 6D compares the genomic environment of proviruses from clones of increasing abundance. The first column of the heat map, which shows the less represented clones (below 1 cell per 10^3^ PBMCs) and was used as reference. The proportion of clones carrying a provirus located next to CpG islands or Refseq genes significantly increased with clone abundance (Chi-squared test for trend, respectively p = 0.0008 and p = 0.001). Although not significant, there was a similar trend for proximity with tRNA genes and tRNA pseudogenes. We conclude that the abundance of a long term established BLV-infected clone is enhanced by the integration of its provirus nearby a transcribed unit.

## Discussion

HTLV-1 induces a persistent infection that is fortunately generally asymptomatic. Nevertheless, in a small proportion of individual and after a long latency, the infection leads to leukemia or lymphoma. These clinical manifestations are correlated with a persistently elevated proviral load and an oligo- or mono-clonal distribution of the infected cells. The same mechanism applies to BLV that induces leukemia or lymphoma in a minor fraction of infected animals. BLV also induces a persistent lymphocytosis in about 40% of the infected cows whereas this is far less common in HTLV-1 infected individuals. In fact, BLV and HTLV-1 are related deltaretroviruses sharing a similar genomic organization and infecting cells of the hematopoietic system (respectively mainly B cells and T cells). In this respect, BLV infection might be useful to address important questions unanswered in the HTLV-1 system. In particular, early infection appears to be critical at determining the different populations of cells (i.e. clones) that will subsequently thrive and expand during pathogenesis [10], [11], [17]. The mechanisms undergoing during this initial period of HTLV-1 infection cannot be addressed because of lack of samples (e.g. when transmission occurs via breast feeding) or due to the absence of systematic screening of populations at risk. In this report, we aimed at identifying the clones created during primary infection (i.e. position of the proviral insertion site in the host genome) and quantifying their abundance (i.e. number of cells per clone). With this objective, we performed high throughput sequencing of proviral insertion sites in BLV-inoculated cows during the early days of infection.

We first show that thousands of clones are generated during primary infection indicating that initial replication occurs through the infectious cycle. None of the newly generated clones massively expanded during this early period revealing a polyclonal pattern of viral expansion and confirming observations made in the BLV-infected sheep model [11] and in squirrel monkey experimentally inoculated with HTLV-1 [10]. A second contribution of this report concerns the genomic sequence of the insertion sites that are strikingly similar in the BLV and HTLV-1 systems. Thirdly, BLV provirus insertion occurs nearby transcribed genomic regions, as reported for HTLV-1 [16], [18], [25]. In fact, most (if not all) retroviruses target a characteristic weak palindromic consensus nucleotide sequence at the site of integration [26], [27] and favor insertion within a particular genomic environment [28]–[30]. These preferences originate from the structure of the pre-integration complex given by the viral integrase [31], [32], its possible cellular partners [33] and from the relative accessibility of the genome [30]. Taken together, our observations thus reinforce the similarities between BLV and HTLV-1.

Therefore, the BLV model can be informative to understand mechanisms of early infection by deltaretroviruses, a period that cannot be easily addressed in the HTLV-1 system as discussed previously due to the logistic and ethical problems of collecting sequential neonatal samples. In BLV-inoculated sheep, the time period delimiting the infectious cycle was estimated to last the first 8 months [11]. Sheep are not natural hosts for BLV and develop leukemia/lymphoma at higher frequencies after shorter latency periods compared to cattle. In our report, we now show that this period generally lasts about 2 months in the bovine species. It is possible that a shorter infectious cycle period also limits the probability of leukemia/lymphoma in the natural host. Indeed, oncogenesis, which is a rare event that occurs in a single or in a limited number of cells, correlates with the number of infected cells. Even though we showed that BLV propagation rapidly shifts to clonal expansion to maintain the bulk of the infected cells, we cannot exclude that infection of new cells by virions has totally been stopped. Because analysis is based on the detection of proviral insertion sites in PBMCs, intermittent burst of virus replication with clearance of the newly infected cells and/or replication in tissues other than the peripheral blood may occur. Alternatively, it is also possible that the infectious cycle is ongoing permanently but that the immune response is sufficiently efficient to destroy all newly infected cells. Also, our method is limited by the number of infected cells present in the collected blood sample and so, the clonality analysis is constrained by the representative sample, making difficult the interpretation of clonal succession data from low proviral load animals such as #21.

Currently, there are no available data pertaining to the early viral replication in HTLV-1 infected patients. Based on this study and previous publications using animal models [10], [11], it is likely that the HTLV-1 infectious cycle is also mainly restricted to the first months post-infection. This prediction has important clinical applications in terms of viral transmission. Since the infectious cycle requires production of virions and reverse transcription, anti-retroviral treatments could be instrumental to avoid mother-to-child transmission during delivery or breast feeding. In several endemic regions, breastfeeding cannot be prohibited because of societal and sanitary reasons. In particular, formula milk requires access to clean water and does not provide passive immunity to local pathogens. Based on this report, we propose that an early and short anti-retroviral treatment soon after birth might reduce HTLV-1 transmission and even possibly limit the long term HTLV-1 load. This option has recently been tested in another retrovirus, HIV that propagates preferentially via neoinfection of lymphocytes. Indeed, HIV-infected patients that initiated cART (combination antiretroviral therapy) during primary infection remained post-treatment controllers with very low viremia for many years [34].

A major contribution of our report is to demonstrate the massive depletion of the early generated clones. In Figure 3, we indeed show that the primary infection period is characterized by the disappearance of the vast majority of clones. Considering the current knowledge in the BLV/HTLV-1 systems [35], [36], it is likely that this clonal selection is due to counter selection by the host immune response. Importantly, the clones carrying a provirus in a transcribed genomic environment were even more susceptible to be destroyed. We speculate that the stronger selection against proviruses located nearby genes might be the result of an increased viral expression and a higher exposure to the host immune system [37]. In this context, HDAC inhibitors have been shown to induce BLV [38], [39] and HTLV-1 expression [40]–[42] and consequently increase the exposure of the infected cells to the host immune system. Remarkably, treatment of STLV-1 infected monkeys with a combination of the HDAC inhibitor valproate and the reverse transcriptase inhibitor AZT induced a persistent decrease of the PVL [43]. Thus, one might consider testing such combination of an antiretroviral drug and an HDAC inhibitor in the early days of the infection.

It has been previously shown that HTLV-1 infected clones are not equal regarding their proliferative potential. Indeed, the abundance of a given clone is enhanced by the integration of its provirus in an actively transcribed area of the genome [16], [18]. We demonstrated here that this mechanism is also present in BLV-infected cows where clones that thrive and proliferate in the long term carry a provirus in a transcribed environment. We should recall that transcriptional activity of the genomic environment of the provirus is inferred from genomic features like CpG islands and genes and so, it is not a direct experimental measurement of the transcription. It thus appears that two opposite forces will act during primary infection and dictate the fate of the long term clonal composition: (1) BLV initially favors integration into genes or promoters and (2) host negative selection disfavors proviruses located next to transcribed regions. The outcome of these two forces will determine the PVL set point value as clonal abundance will benefit from carrying a provirus in transcribed regions but will concomitantly also be reduced by the immune response. Infected hosts able to more efficiently eliminate clones carrying a provirus integrated nearby transcribed units will thus have lower PVLs. Differences in immune response efficiencies have been identified in BLV-infected animals as well as in HTLV-1 infected individuals [44]–[47]. These differences translate into disease susceptibilities and were related at least partially to the genotype of the host [48]–[53]. Our preliminary data show that negative selection against clones carrying a provirus nearby transcribed regions is more efficient in animals that will subsequently present low proviral load set point (data not shown).

At a first glance, the observation that clone abundance positively correlates with provirus proximity with transcribed units appears to conflict with the extremely low levels of viral expression measured in vivo [54], [55]. Indeed, structural viral transcripts from peripheral blood lymphocytes or tumors can only be amplified by the means of very sensitive techniques such as in situ hybridization or RT-PCR. With progression from persistent lymphocytosis to leukemia, BLV expression levels even tend to decrease [56]. In tumors, BLV proviruses can be completely silent regarding viral messenger RNAs albeit carrying an intact genomic sequence [57]. Similarly, 5′-LTR directed transcription of HTLV-1 can be completely abrogated in leukemic clones by methylation and by deletion or mutation of viral genes [58]–[60]. Nevertheless, proviral silencing of BLV and HTLV-1 appeared to be selective, leaving some specific transcript untouched. This is the case for HBZ (HTLV-1 b-ZIP factor) that remains strongly expressed in ATLL cells [61]. Recently, BLV has been shown to produce microRNAs that are also actively transcribed in BLV malignant cells [62]. Mechanistically, the silencing of BLV structural genes can be achieved by repressive histone marks deposited onto the promoter [63] without interfering with pol III dependent transcription of the BLV microRNAs [62], [64]. Consistently, we detected constitutive expression of BLV-miR-B4-3p despite very low levels of structural viral transcripts (data not shown). In this context, it is important to note that BLV favors pol III transcribed regions (tRNA genes and pseudogenes) upon insertion (Figure 4C). Furthermore, abundance of these established clones benefits from a provirus inserted in pol III transcribed regions (Figure 6). We might speculate that this genomic environment could in turn favor BLV microRNA expression. We propose that the abundance of successful clones benefit from the transcriptional activity of the genomic region surrounding the provirus to maintain robust expression of BLV microRNAs. Alternatively, BLV insertion nearby pol III regulated genes such as tRNAs, 5S rRNA and other small RNAs may affect their activity and modify the cell metabolism. In particular, pol III directed transcription of housekeeping genes is a key parameter of cell growth and replication.

In summary, we have characterized BLV clonality during primary infection using high throughput sequencing of the proviral insertion sites in 7 sequential samples of 5 BLV-inoculated cows and as well as in long term infected animals. We demonstrate that BLV proviruses initially integrate into transcribed regions of the genome but are massively depleted later on. This mechanism may have important outcomes for HTLV-1 prevention and treatment.

## Materials and Methods

### Experimental setting of BLV infection

Cows were experimentally infected with the wild-type BLV strain 344. Two 15 cm-diameter dishes containing subconfluent Hela cells were transfected with 80 µg of plasmid pBLV344 containing the BLV provirus, recovered in 5 ml PBS at day 3 and injected subcutaneously. The presence of anti-BLV antibodies was determined using a competitive ELISA test (IDEXX Leukosis Blocking Ab Test). Peripheral blood mononuclear cells (PBMCs) were isolated by Ficoll Hypaque density gradient centrifugation (Sigma-Aldrich), washed and cryopreserved in fetal calf serum with 10% DMSO (Sigma-Aldrich).

### Ethics statement

Animal experimentations were conducted in accordance with national and international guidelines for animal care and use described in the Manual for use and care of experimental animals emitted by INTA. Handling of cows and experimental procedures were reviewed and approved by INTA's Institutional Committee for Care and Use of Experimental Animals (CICUAE-INTA) under protocol number 35/2010.

### BLV proviral load measurement

DNA was extracted from PBMCs using DNeasy Blood and Tissue kit (Qiagen). BLV DNA was PCR amplified using pol gene sequence-specific primers 5′-GAAACTCCAGAGCAATGGCATAA-3′ and 5′-GGTTCGGCCATCGAGACA-3′. As reference for genomic DNA, β-actin was amplified with oligonucleotides 5′-TCCCTGGAGAAGAGCTACGA-3′ and 5′-GGCAGACTTAGCCTCCAGTG-3′. Three dilutions of DNA (100 ng, 33 ng and 11 ng) were amplified by real-time quantitative PCR in a Roche light cycler using MESA green master mix (Eurogentec). The thermal protocol used started with a 95°C 5 min denaturation step; then 45 cycles as follows (95°C 15 sec, 60°C 20 sec, 72°C 40 sec) and terminated with a melting curve. PCR efficacies were calculated for each sample using the three dilutions. Standard curves were generated using PCR4topo vectors (Life Technologies) containing the corresponding pol or actin amplicon. Proviral load was calculated, as an average of the three dilutions, from the number of proviral copies divided by half of the number of actin copies and expressed as number of proviral copies per 100 of PBMCs.

### Selective amplification and quantification of BLV proviral insertion sites

Ten micrograms of genomic DNA extracted from PBMCs were sheared by sonication with the *Diagenode* Bioruptor instrument using the following protocol (15 sec ON, 90 sec OFF, 4 cycles in a 4°C water bath). DNA ends were then end-repaired using 15 units of T4 DNA polymerase (New England Biolabs), 5 units of DNA polymerase I Klenow fragment (NEB), 50 units of T4 polynucleotide kinase (NEB) and 0.8 mM of dNTP (Sigma) in T4 DNA ligase buffer (NEB) at 20°C during 30 min. DNA was then cleaned using a Qiaquick PCR purification kit (Qiagen) and eluted in 64 µl of EB buffer. Addition of an adenosine at the 3′ ends of the DNA was performed by adding 0.2 mM of dATP (Sigma) and 15 units of Klenow Fragment 3′ to 5′ exo- (NEB) in NEB2 buffer (NEB) at 37°C for 30 min. DNA was then cleaned using a Qiaquick PCR purification kit and eluted in 40 µl of EB. One hundred pmol of a partially double stranded DNA linker was ligated to the DNA ends using a Quick ligation kit (NEB). Twenty four different linkers were designed, each one with a specific 8 bp tag (see Primer list in Text S1) to allow multiplexing of DNA samples during the sequencing. DNA was cleaned using a Qiaquick PCR purification kit and eluted in 60 µl of EB. The 60 µl of ligated product was then split into 3 aliquots of 20 µl and each aliquot was used in a separate PCR1 reaction (see Figure S1). For each PCR reaction, 20 µl of ligated product was mixed with 0.2 mM of dNTP (Sigma), 50 pmol of BLV_LMPCR1 primer (binds BLV LTR), 10 pmol of VU primer (which anneals to the strand of the Vectorette Unit linker generated by the amplification from BLV_LMPCR1), 1 unit of Phusion DNA polymerase in High Fidelity buffer (Finnzyme, NEB). The following thermal protocol was used: denaturation for 30 sec at 98°C; then 30 cycles (5 sec at 98°C, 10 sec at 62°C, 30 sec at 72°C); followed by 10 min at 72°C; and finally cooled at 4°C. The 3 PCR1 products, derived from the same sample were then pooled. The DNA was cleaned using a Qiaquick PCR purification kit and eluted in 150 µl of EB. To perform PCR2, 1 ul of the cleaned PCR1 product was mixed with 0.2 mM of dNTP (Sigma), 25 pmol of P5_BLV_LMPCR2 primer binding the BLV LTR), 25 pmol of P7 primer binding the linker, 1 unit of Phusion DNA polymerase in High Fidelity buffer (NEB). The following thermal protocol was used: 98°C for 30 sec; 30 cycles (5 sec at 98°C, 10 sec at 62°C, 30 sec at 72°C); 10 min at 72°C; 4°C until user stops. DNA was then cleaned using a Qiaquick PCR purification kit and eluted in 50 µl of EB. A library was constructed by pooling the different PCR2 products (each one possessing a specific tag). Quantification of the libraries was made by qPCR using primers P5 and P7 (see Primer list in Text S1) and a MESA green master mix (Eurogentec) in a Roche light cycler 480 instrument. Three dilutions of the library (200 pg, 66 pg and 22 pg) were amplified. Standard curves were generated using a library quantified on a titration flow cell previously run on a Genome Analyzer II (Illumina). Stock libraries were diluted down to 8 pM and clustered on the flow cell. Paired-end reads (read1 and read2 each 50 bp) plus a 8 bp tag read (read 3) were acquired on a GA II or a Myseq Illumina instrument. Read 1 and read 2 were mapped against the bovine genome (build Bos_taurus_UMD_3.1/bosTau6) and the proviral insertion site and the shear site were deduced.

“Sister cells” are cells where the BLV provirus is inserted at the same site in the cellular genome and a “clone” is a population of sister cells. For each unique insertion site, the number of amplicons of different length (i.e. different shear sites) give an estimate of the number of sister cells of that infected clone [65]. The absolute abundance of a given clone *i* (number of cells per 100 PBMCs) was calculated from the number of sister cells and the measurement of the proviral load as follows:
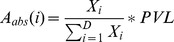
where *X_i_* is the number of sister cells of the *i*th clone, *D* the number of observed clones and *PVL* the proviral load.

The relative abundance of a given clone *i* (in percent of the proviral load) was expressed as follows:
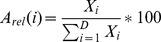



### Oligoclonality index

To measure the clonality of the infected cell population, i.e. the non-uniformity of the clone abundance distribution, we used the oligoclonality index based on the Gini coefficient [66]. Oligoclonality index ranges from 0 (all the BLV-infected clones having the same abundance, i.e. perfect polyclonality) to 1 (only one BLV-infected clone constitutes the total proviral load, i.e. perfect monoclonality). Details of the calculations are given in supplemental file Text S1 (Supplemental Materials and Methods).

### Similarity index

To measure the likeliness or overlap between two populations of BLV-infected cells, we calculated a similarity index. This number ranges from 0 to 1, with 0 indicating that no clones are shared between the two populations and 1 corresponding to a complete identity (all the clones present in population 1 were also present in population 2 and *vice versa*). Because this index takes clone abundance into account, populations that contain the same clones but have different clone abundance will have an index value of less than 1. Similarity indexes were calculated between 2 successive time points separated by two weeks. Details of the calculations are given in supplemental file Text S1 (Supplemental Materials and Methods).

### Genetic environment around the proviral insertion site and generation of random sites

Favored genomic DNA sequences for BLV or HTLV-1 integration were represented using WebLogo 3 [67]. Genomic annotations flanking the proviral insertion sites were retrieved using Galaxy [68] which is a web-based genome analysis tool. DNA sequences (read 1 and read 2 like sequences) from 100,000 random sites in the bovine genome were generated using Galaxy and back-aligned to the bovine genome using the same pipeline to eliminate any potential bias due to alignment limitations.

### Statistics

Statistical tests were performed using GraphPad Prism, Microsoft Excel and R softwares. The symbol *** was used when p<0.001, ** when p<0.01, * when p<0.05, NS (Non Significant) when p>0.05 and NA for Non Applicable.

## Supporting Information

Text S1 The supporting file Text S1 contains supporting tables S1 and S2, supporting material and methods and supporting figure S1.
(PDF)
