# Supplementary material for: Massive Depletion of Bovine Leukemia Virus Proviral Clones Located in Genomic Transcriptionally Active Sites during Primary Infection
Source: PLoS Pathog. 2013 Oct 3;9(10):e1003687. doi: 10.1371/journal.ppat.1003687 (PMC3789779; doi:10.1371/journal.ppat.1003687)
Supplement: Text S1 — The supporting file Text S1 contains supporting tables S1 and S2, supporting material and methods and supporting figure S1. (PDF) [file ppat.1003687.s001.pdf]

Supplemental Table

Table S1: number of clones detected per animal and per time point during primary infection

animal ID number	time point of the blood sample (day post inoculation)	no of clones detected	total no of unique clones detected
#21	16	62	
#21	30	59	
#21	43	30	
#21	57	78	
#21	68	22	
#21	252	15	
#21	267	11	
#21			264
#23	16	6	
#23	30	2266	
#23	43	2307	
#23	57	1537	
#23	68	793	
#23	252	5	
#23	267	5	
#23			5775
#31	16	11	
#31	43	5520	
#31	57	1861	
#31	68	1113	
#31	252	13	
#31	267	9	
#31			7669
#492	16	14	
#492	30	714	
#492	43	788	
#492	57	729	
#492	68	733	
#492	252	710	
#492	267	578	
#492			2281
#535	16	3	
#535	30	2872	
#535	43	4915	
#535	57	3833	
#535	68	3056	
#535	252	1641	
#535	267	768	
#535			12906

NB: The last column shows the total number of unique clones detected within each animal. Clones detected at several time points were scored only once.

Table S2: number of clones detected in long term infected animals (BLV-positive for more than 2 years)

animal ID number	no of clones detected
#5	6824
#6	1544
#36	1529
#46	1284
#144	14
#358	23
#359	89

Supplemental Materials and Methods

Oligoclonality index

The clonality of a clone population was determined by a measure of the non-uniformity of the frequency distribution of clone abundance.

The oligoclonality index (OCI) is based on the Gini coefficient [1] and is calculated as

$$OCI = 2 * \left(\sum_{k=1}^{D} \frac{P_k}{D} - 0.5 \right)$$

Where

X_i the number of sister cells for a given clone i

D the total number of clones identified in the sample

$N = \sum_{i=1}^{D} X_i$ the sum of the numbers of sister cells for all the clones

$p_i = \frac{X_i}{N}$ the relative abundance of each clone

$P_k = \sum_{i=1}^{i=k} p_i$ the cumulative relative abundance of clone ranked in decreasing order of abundance

Similarity index

The overlap or similitude between two successive populations of BLV-infected cells was evaluated by the Morisita's abundance-based similarity index [2].

The similarity index (SI) is calculated as

$$SI = \frac{2\sum_{i=1}^{D_{12}} \frac{X_i}{n}\frac{Y_i}{m}}{\sum_{i=1}^{D_1}\left(\frac{X_i}{n}\right)\left(\frac{X_i-1}{n-1}\right)+\sum_{i=1}^{D_2}\left(\frac{Y_i}{m}\right)\left(\frac{Y_i-1}{m-1}\right)}$$

where

(X_i, Y_i) : number of sister cells of the ith clone in the two populations

$$n = \sum_{i=1}^{D_1} X_i$$

$$m = \sum_{i=1}^{D_2} Y_i$$

D_1 : number of observed clones in population 1 (time point 1)

D_2 : number of observed clones in population 2 (time point 2)

D_{12} : number of observed shared clones in the two populations

The similarity indices were computed using SPADE software [3].

Primer list

BLV_LMPCR1 primer

5’ CTCTCTCTTGCCTCCTGACC

VU primer

5’ TCATGATCAATGGGACGATCA

P5_ BLV_LMPCR2 primer

5’ AATGATACGGCGACCACCGAGATCTACACGCGCTTGTTTCCTGTCTTACTT

P5 primer

5’ AATGATACGGCGACCACCGAGAT

P7 primer

5’ CAAGCAGAAGACGGCATACGA

Sequencing primer read1

5’ CTCTCTCCTTCGGCGCCCTCTAGCGGCCAGGAGAGACCG

Sequencing primer read2

5’ CGGTCTCGGCATTCCTGCTGAACCGCTCTTCCGATCT

Sequencing primer 8bp tag

5’ GATCGGAAGAGCGGTTCAGCAGGAATGCCGAGACCG

Vectorette_Unit_upper_arm

5'p-GATCGGAAGAGCGAAAAAAAAAAAAA

Vectorette_Unit_lower arm with different tag

VU1 <u>ATCACGGG</u>

5'TCATGATCAATGGGACGATCACAAGCAGAAGACGGCATACGAGAT<u>CCCGTGAT</u>CGGTCTCGGCATTCCTGCTG
AACCGCTCTTCCGATCT

VU2 TAAAGCAT

5'TCATGATCAATGGGACGATCACAAGCAGAAGACGGCATACGAGATATGCTTTACGGTCTCGGCATTCCTGCTG AACCGCTCTTCCGATCT

VU3 GCAGTCCT

5'TCATGATCAATGGGACGATCACAAGCAGAAGACGGCATACGAGATAGGACTGCCGGTCTCGGCATTCCTGCTG AACCGCTCTTCCGATCT

VU4 CAGCTACG

5'TCATGATCAATGGGACGATCACAAGCAGAAGACGGCATACGAGATCGTAGCTGCGGTCTCGGCATTCCTGCTG AACCGCTCTTCCGATCT

VU5 AATTCCGG

5'TCATGATCAATGGGACGATCACAAGCAGAAGACGGCATACGAGATCCGGAATTCGGTCTCGGCATTCCTGCTG AACCGCTCTTCCGATCT

VU6 AGCTAGCC

5'TCATGATCAATGGGACGATCACAAGCAGAAGACGGCATACGAGATGGCTAGCTCGGTCTCGGCATTCCTGCTG AACCGCTCTTCCGATCT

VU7 CGCCTACG

5'TCATGATCAATGGGACGATCACAAGCAGAAGACGGCATACGAGATCGTAGGCGCGGTCTCGGCATTCCTGCTG AACCGCTCTTCCGATCT

VU8 ACCCGATT

5'TCATGATCAATGGGACGATCACAAGCAGAAGACGGCATACGAGATAATCGGGTCGGTCTCGGCATTCCTGCTG AACCGCTCTTCCGATCT

VU9 GCTAAGCC

5'TCATGATCAATGGGACGATCACAAGCAGAAGACGGCATACGAGATGGCTTAGCCGGTCTCGGCATTCCTGCTG AACCGCTCTTCCGATCT

VU10 GGTATATA

5'TCATGATCAATGGGACGATCACAAGCAGAAGACGGCATACGAGATTATATACCCGGTCTCGGCATTCCTGCTG AACCGCTCTTCCGATCT

VU11 TGTGTGTG

5'TCATGATCAATGGGACGATCACAAGCAGAAGACGGCATACGAGATCACACACACGGTCTCGGCATTCCTGCTG AACCGCTCTTCCGATCT

VU12 GTGTACAC

5'TCATGATCAATGGGACGATCACAAGCAGAAGACGGCATACGAGATGTGTACACCGGTCTCGGCATTCCTGCTG AACCGCTCTTCCGATCT

VU13 TGGACACA

5'TCATGATCAATGGGACGATCACAAGCAGAAGACGGCATACGAGATTGTGTCCACGGTCTCGGCATTCCTGCTG AACCGCTCTTCCGATCT

VU14 AAAGATCG

5'TCATGATCAATGGGACGATCACAAGCAGAAGACGGCATACGAGATCGATCTTTCGGTCTCGGCATTCCTGCTG AACCGCTCTTCCGATCT

VU15 CCAAGCAT

5'TCATGATCAATGGGACGATCACAAGCAGAAGACGGCATACGAGATATGCTTGGCGGTCTCGGCATTCCTGCTG AACCGCTCTTCCGATCT

VU16 CCGGCAAA

5'TCATGATCAATGGGACGATCACAAGCAGAAGACGGCATACGAGATTTTGCCGGCGGTCTCGGCATTCCTGCTG AACCGCTCTTCCGATCT

VU17 GGGTACAT

5'TCATGATCAATGGGACGATCACAAGCAGAAGACGGCATACGAGATATGTACCCCGGTCTCGGCATTCCTGCTG AACCGCTCTTCCGATCT

VU18 CAAACACG

5'TCATGATCAATGGGACGATCACAAGCAGAAGACGGCATACGAGATCGTGTTTGCGGTCTCGGCATTCCTGCTG AACCGCTCTTCCGATCT

VU19 TTGGATTT

5'TCATGATCAATGGGACGATCACAAGCAGAAGACGGCATACGAGATAAATCCAACGGTCTCGGCATTCCTGCTG AACCGCTCTTCCGATCT

VU20 CTGATACA

5'TCATGATCAATGGGACGATCACAAGCAGAAGACGGCATACGAGATTGTATCAGCGGTCTCGGCATTCCTGCTG AACCGCTCTTCCGATCT

VU21 TCGAAGCT

5'TCATGATCAATGGGACGATCACAAGCAGAAGACGGCATACGAGATAGCTTCGACGGTCTCGGCATTCCTGCTG AACCGCTCTTCCGATCT

VU22 TTAGCGCG

5'TCATGATCAATGGGACGATCACAAGCAGAAGACGGCATACGAGATCGCGCTAACGGTCTCGGCATTCCTGCTG AACCGCTCTTCCGATCT

VU23 AAGGAGAT

5'TCATGATCAATGGGACGATCACAAGCAGAAGACGGCATACGAGATATCTCCTTCGGTCTCGGCATTCCTGCTG AACCGCTCTTCCGATCT

VU24 GATAAGCC

5'TCATGATCAATGGGACGATCACAAGCAGAAGACGGCATACGAGATGGCTTATCCGGTCTCGGCATTCCTGCTG AACCGCTCTTCCGATCT

References

1. Gini (1914) Sulla misura della concentrazione e della variabilita dei caratteri: Transactions of the Real Istituto Veneto di Scienze.
2. Chao A, Chazdon RL, Colwell RK, Shen TJ (2006) Abundance-based similarity indices and their estimation when there are unseen species in samples. Biometrics 62: 361-371.
3. Chao A, Shen TJ (2010) Program SPADE (Species Prediction And Diversity Estimation). Program and User's Guide published at http://chao.stat.nthu.edu.tw.

Figure S1

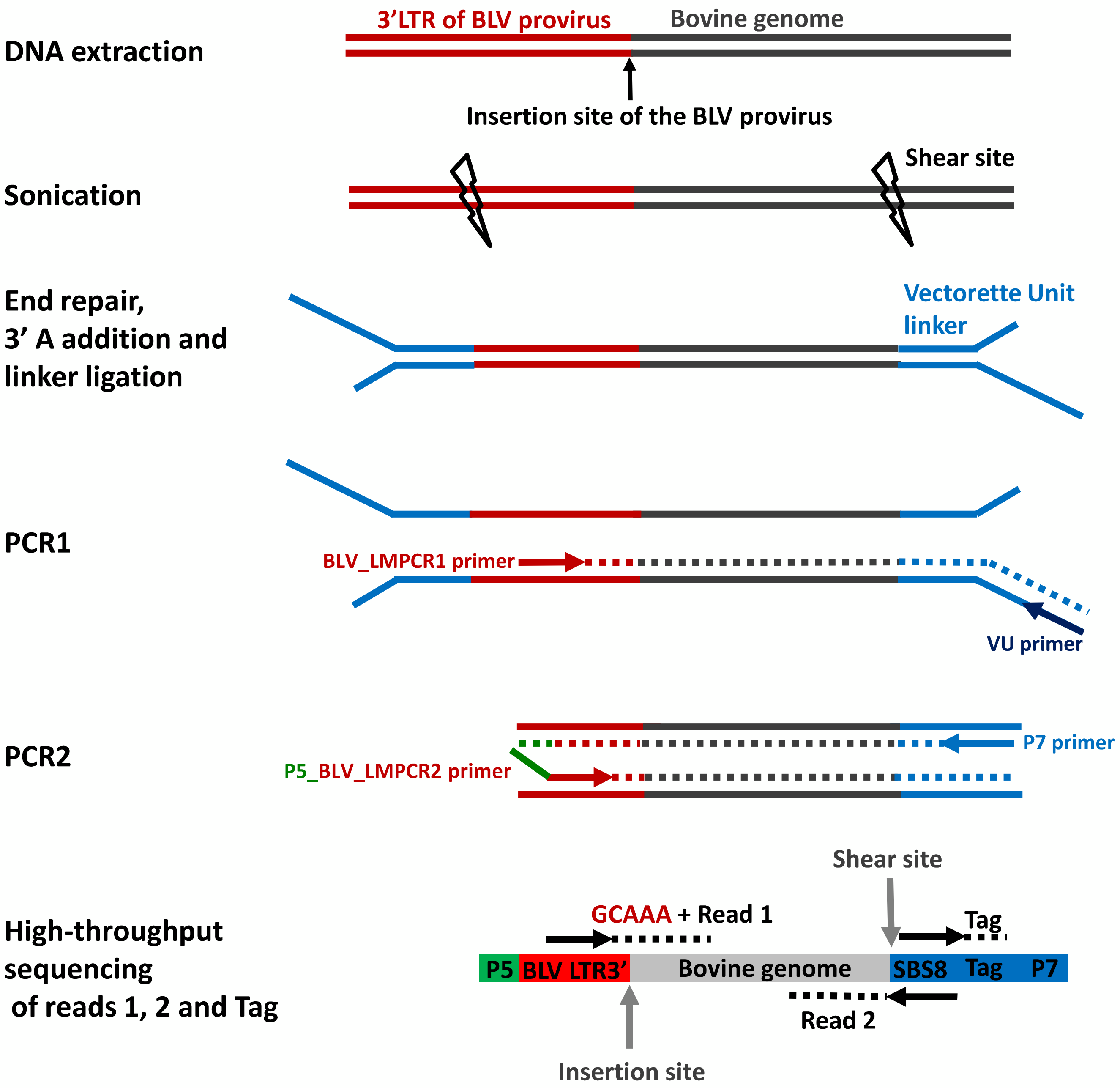

Figure S1: Mapping of BLV proviral insertion sites and quantification of clone abundance. DNA was extracted from PBMCs and sheared by sonication. DNA ends were repaired, polyadenylated and ligated to a tagged double-stranded linker with mismatched ends (Vectorette Unit). Nested PCR was performed between the end of the BLV long terminal repeat and the linker. A paired-end read (read 1 and read 2) plus a tag read were acquired on a high throughput sequencer. Read 1 and read 2 were then mapped on the bovine genome.
